# Rasch Analysis and Interval‐Level Scaling of the Positive and Negative Affect Schedule (PANAS) Across Cultures

**DOI:** 10.1002/ijop.70230

**Published:** 2026-06-08

**Authors:** Peter Adu

**Affiliations:** ^1^ Division of Social and Cultural Psychiatry, Department of Psychiatry McGill University Montreal Canada; ^2^ School of Health, Wellington Faculty of Health Victoria University of Wellington Wellington New Zealand

**Keywords:** negative affect, positive affect, Rasch analysis, reliability, validity

## Abstract

Affective state investigation requires precise measurement that satisfies parametric statistical assumptions for reliable and valid cross‐data comparisons. The psychometric properties of the 20‐item Positive and Negative Affect Schedule (PANAS) are satisfactory, but provide scores on an ordinal scale, which could be unsuitable for parametric statistics. The present study employed the Rasch model to assess the psychometric statistics of the PANAS, enhancing the scale's precision using community samples from four countries. I analysed responses from a randomly selected sample of 1000 individuals (250 from each country) out of a total sample of 1822 recruited from Germany (475), Ghana (523), India (411), and New Zealand (413). The analyses indicated that both the positive affect and negative affect subscales demonstrated satisfactory model fit after applying the testlet creation approach. Each subscale reflected a clear, single underlying construct with strong reliability and structural validity. Both scales also functioned equivalently across demographic groups, suggesting that the items measured affective states consistently, regardless of participants' sociodemographic backgrounds. The scales further exhibited strong convergent and discriminant validity. The study developed an algorithm to convert ordinal scores to interval data, enhancing precision and validity in parametric analyses.

## 1 Introduction

Affective states significantly influence overall health and well‐being. They can be broadly categorised as positive (e.g., happiness) or negative (e.g., sadness), shaping emotional responses and behavioural patterns. These states refer to mood states that persist over extended periods rather than short‐lived emotional responses (Watson et al. 1988). Positive affect is particularly valuable as it is linked to better health outcomes and behaviours (e.g., Adu et al. 2025). Research has shown that individuals with positive emotions are more likely to engage in physical activities, maintain a healthy diet, and adopt more beneficial lifestyle habits (Adu 2025). Positive affect was also associated with reduced pain, lower morbidity, and increased longevity, particularly among the aged population (Pressman and Cohen 2005). On the other hand, negative affect has consistently been found to influence poor health outcomes. For example, negative affect was significantly related to risky health behaviours, such as smoking among adolescents (Young et al. 2019). Negative affect mediates the impact of family and occupational stress on both psychological distress and physical health, and it is also most strongly linked to poorer oral health‐related quality of life (Brennan et al. 2006).

Perhaps even more concerning is the impact of affect in the era of the COVID‐19 pandemic. Adu (2025) found a positive relation between positive affect and COVID‐19 vaccination attitudes among participants recruited from Germany, Ghana, India, and New Zealand. Other studies have provided evidence regarding the relation between affective states and vaccination. For example, Klasko‐Foster et al. (2020) found a positive correlation between positive affect and the human papillomavirus vaccine in the USA. A study also identified that negative affect inversely predicts life satisfaction in individualist cultures compared to collectivist cultures (Kuppens et al. 2008), indicating the influence of culture on affective states in determining well‐being. The emerging evidence demonstrates that cross‐cultural assessment of valid measures, such as those aimed at in this study, is important for evaluating affective states. This is particularly important for facilitating the overall well‐being of individuals across countries.

For decades, the 20‐item self‐reported Positive and Negative Affect Schedule (PANAS) has been instrumental in evaluating affects internationally (Roemer and Medvedev 2023). The 2‐factor PANAS, originally developed by Watson et al. (1988), has demonstrated satisfactory validity and reliability across different cultures. The reliability coefficients for the PANAS have been reported to range from 0.83 to 0.91 for the full scale and from 0.70 to 0.93 for the subscales (Roemer and Medvedev 2023). This scale is also available in various languages (e.g., Italian, French, German, Arabic, Spanish) and includes subscales for both Positive Affect (PA) and Negative Affect (NA).

Notably, this evidence from the traditional Confirmatory Factor Analysis (CFA) based on Classical Test Theory (CTT) is limited by its use of ordinal scores. These scores have drawbacks such as low precision, proneness to producing misleading correlations, and unsuitability for use in parametric statistical analyses (Boone 2016). These limitations are particularly evident in studies examining the factor structures of the PANAS, where some research using bifactor models has reported three factors instead of the original two (Roemer and Medvedev 2023). Thus, in CFA, the standardisation process of ordinal data can distort factor loadings because its implied assumption of equal intervals between response categories may not accurately reflect true differences between categories, leading to potential inaccuracies in the analysis of ordinal data (Boone 2016).

Moreover, the use of the Rasch model, rooted in the Item Response Theory (IRT), tends to override the limitations of the CFA by providing robust means for assessing the psychometric parameters of scales. The Rasch model transforms raw ordinal data into a linear measure (i.e., logit scale) representing the latent trait on a continuous scale for analysis with parametric statistical tests (Boone 2016). According to the principles of Rasch model, intervals between adjacent rating categories of items can be unequal, and their pattern may differ from item to item, reflecting varying levels of item difficulty (Boone 2016). Hence, Rasch model assumes that the probability of an individual endorsing a specific response category for an item depends on the difference between the person's ability and the item's difficulty (Boone 2016). Rasch analysis is also often considered less sample‐dependent because it focuses on the item‐person fit to the model, allowing for more stable item parameters that can be generalised across different samples. Conversely, CFA estimates are typically more sample‐dependent because CFA relies on the covariance structure of the data, which can vary with different samples (Boone 2016).

Notwithstanding, while Rasch analysis offers some clear advantages over CTT, particularly in transforming ordinal data into interval measures and ensuring item‐level invariance, it is not without limitations. For instance, while testlets help retain the content validity of a scale, their use in Rasch analysis can introduce interpretational ambiguity and artificial dimensionality if not applied carefully. Testlet formation may sometimes obscure distinctions between related constructs or create dependencies that alter the scale's internal structure (Marais and Andrich 2008). For example, forming testlets based solely on item content can be misleading, as semantic meanings may vary across cultural contexts (Adu et al. 2025; Christensen et al. 2017). Therefore, while Rasch modelling enhances psychometric precision, it requires thoughtful application and transparent reporting to mitigate these potential drawbacks.

The use of Rasch model for the PANAS was limited to few studies (Medvedev et al. 2023; Peter et al. 2016; Pires et al. 2013) in the literature. While some of these studies have reported satisfactory model fit indices for the PANAS, others have failed to confirm unidimensionality, with item misfit emerging as a recurring issue, particularly within the PA subscale. Such discrepancies in the PANAS, particularly at the item‐level diagnosis analysis, offer important insights into the scale's cross‐cultural robustness. These findings suggest that aggregated analyses, such as CFA, may overlook nuances in how affective constructs function across cultures. They may fail to capture valence structure differences that exist between cultural orientations such as collectivism and individualism. For instance, in individualist cultures (e.g., Germany and the US), positive and negative emotions are typically viewed as independent, as individuals can feel happy or sad at different times, and the pursuit of positive emotion and avoidance of negative emotion are seen as natural.

Conversely, in collectivist cultures (e.g., Ghana and China), emotions tend to be experienced more interdependently or in balance; individuals may simultaneously experience happiness and sadness, reflecting cultural values that emphasise emotional moderation and social harmony (Bagozzi et al. 1999; Tsai et al. 2006). Additionally, these Rasch‐based studies have also predominantly focused on Swiss clinical sample and specific populations from Brazil and New Zealand, which restricts the scope of cross‐cultural comparison of the PANAS regarding Rasch findings. Only one study provided a conversion table for transforming ordinal data to interval‐level data for the PANAS (Medvedev et al. 2023), which is essential step in Rasch application for measures (Boone 2016). These limitations impact the generalizability and robustness of the PANAS, highlighting a significant gap in the literature and underscore the need for more comprehensive studies using diverse samples. Consequently, the current study assessed the psychometric statistics of the 20‐item PANAS using a community sample from Ghana, Germany, India, and New Zealand employing the Rasch model to enhance the scale's precision. Although the English and German versions of the scale have been used in these countries, its validation within these contexts remains insufficiently established. Our focus was on investigating reliability, structural validity, convergent and discriminant validities, while also exploring measurement invariance.

The study expected a statistically significant strong positive relation between PA scores and related measures of compassion towards others, optimism, and self‐compassion (Förster and Kanske 2022). Conversely, the study hypothesised a positive relation between NA scores and psychological distress (Guan et al. 2021). These correlations were expected to establish convergent validity for the subscale (i.e., the scale is positively associated with other theoretically similar constructs). Additionally, the study aimed to demonstrate weak to zero correlations (e.g., *r* = ≤ 0.30; Hemphill 2003) between PA and psychological distress, as well as between NA and measures of compassion towards others, optimism, and self‐compassion, to support the discriminant validity of the scale (i.e., indicating that it measures constructs distinct from related but theoretically different domains). Such results will further identify both protective and risk factors for affective states.

## 2 Methods

### 2.1 Participants

For the purposes of the Rasch analysis, this study used Microsoft Excel to randomly select 1000 (i.e., 250 from each country) participants from the total sample of 1822 individuals recruited from Germany (475), Ghana (523), India (411), and New Zealand (413). The selected sample mirrored the characteristics and diversity of the entire sample. The selection of the sample size was based on the recommended requirements for Rasch analysis, particularly when using Rasch Unidimensional Measurement Model (RUMM; Hagell and Westergren 2016), which suggests a maximum required sample size of about 500 participants. This final sample size was chosen to balance the benefits of having a larger sample with the potential issue of the *χ* ^*2*^ test's tendency to show statistical significance in larger samples, which could overemphasise minor data misfits with minimal practical relevance (Pelton 2002). The data collection took place in mid‐2022 and is part of a larger dataset examining the interplay between psychological variables and vaccination attitudes, using various methodological and conceptually meaningful approaches (e.g., Adu et al. 2023). The age of participants ranged from 18 to 80 years in India (*M*_age_ *=* 26.14; SD = 8.57), 18–89 years in New Zealand (*M*_age_ = 46.35; SD = 18.07), 18 to 63 years in Ghana (*M*_age_ = 29.48; SD = 5.69), and 18–87 years in Germany (*M*_age_ = 44.09; SD = 5.57). The randomly selected participants differed statistically significant in terms of levels of education (*χ* ^*2*^ (63) = 363.68, *p* < 0.001), sex (*χ* ^2^ (36) = 92.91, *p* < 0.001), and age (*χ* ^*2*^ (18) = 360.45, *p* < 0.001), across the countries.

### 2.2 Procedure

The current cross‐sectional online study was approved by the author's institutional Human Research Ethics Committee, and it aligns with the Declaration of Helsinki, which outlines fundamental ethical principles for health research involving the use of human participants. Due to resource constraints, data collection across the participating countries required adapting different recruitment strategies depending on each country's context, available infrastructure, and participant accessibility. Consequently, data collection in Ghana and India relied on the researcher's networks, utilising platforms such as Facebook, WhatsApp, Twitter, Instagram, and email, employing convenience sampling through a snowballing technique via selectsurvey.net software, with no incentives for participants. In contrast, data collection in New Zealand and Germany was managed by Qualtrics, a data collection company, with participants receiving a small monetary compensation (e.g., NZ$5 or a chance to enter a draw). Participants first provided demographic information before completing the main survey, which took about 15 min. The survey was administered in the official languages of instruction of the participating countries: English for respondents in Ghana, India, and New Zealand, and German for those in Germany. Despite the linguistic diversity in Ghana and India, English is widely employed as the primary language for academic and research activities (e.g., Adu et al. 2021).

### 2.3 Measures

#### 2.3.1 Positive and Negative Affect Schedule

The 20‐item Positive and Negative Affect Schedule (PANAS; Watson et al. 1988) was used to assess positive and negative affect. Each of the two subscales consists of 10 adjectives representing either PA or NA. Participants rated each adjective on a 5‐point Likert scale, ranging from 1 = ‘*very slightly*’ to 5 = ‘*extremel*y’. Examples of adjectives measuring PA include ‘interested,’ ‘strong,’ and ‘proud’; while NA includes ‘anger’, ‘fear’, and ‘sadness’. In the present study, the internal consistency of all scales was assessed using the Rasch‐based Person Separation Index (PSI). While the internal consistency coefficients for the PA subscale were excellent (PSI = 0.91; *M* = 31.40, SD = 7.40), the NA subscale showed very good reliability (PSI = 0.81; *M* = 23, SD = 8.70).

#### 2.3.2 Compassion Towards Others

The 5‐item Santa Clara Brief Compassion Scale (SCBCS; Hwang et al. 2008) was used to measure compassion towards others. Responses are rated on a 7‐point Likert scale, ranging from 1 = ‘*not at all true of me* to’ 7 = ‘*very true of me*’. Example of an item found on the scale is ‘I tend to feel compassion for people, even though I do not know them’. The reliability was found to be very good (PSI = 0.81; *M* = 24.10, SD = 7.00).

#### 2.3.3 Life Orientation Test

This study employed the Life Orientation Test Revised (LOT‐R; Glaesmer et al. 2008; Scheier et al. 1994) to measure dispositional optimism using a 10‐item scale, rated on a 5‐point Likert scale ranging from 0 = ‘*strongly disagree*’ to 4 = ‘*strongly agree*’. For instance, a positively worded item on this scale is ‘In uncertain times, I usually expect the best’. The scale demonstrated acceptable reliability (PSI = 0.73; *M* = 16.10, SD = 3.62; Table 2).

#### 2.3.4 Self‐Compassion

The 12‐item Self‐Compassion Scale‐Short Form (SCS‐SF; Raes et al. 2011) was utilised to measure self‐compassion. This scale uses a 5‐point Likert‐scale response format: 1 = ‘*Almost Neve*r’ to 5 = ‘*Almost Always*’. An example of positively worded item on this scale is ‘I try to be understanding and patient towards those aspects of my personality I don't like’. The reliability of the scale was acceptable (PSI = 0.71; *M* = 31.40, SD = 7.40).

#### 2.3.5 Psychological Distress

The 21‐item Depression Anxiety Stress Scale (DASS‐21; Lovibond and Lovibond 1995), rated on a 4‐point Likert‐scale response option: from 0 = ‘Did not apply to me at all’ to 3 = ‘Applied to me very much’ was utilised to measure psychological distress. Each subscale contains 7 items. An example of items on this scale is: depression (‘I couldn't seem to experience any positive feeling at all’), anxiety (‘I was aware of dryness in my mouth’), and stress (‘I found it hard to wind down’). The reliability coefficient was very good for the overall scores of the DASS‐21 (PSI = 0.87; *M* = 30.80, SD = 18.00).

### 2.4 Statistical Analysis

#### 2.4.1 Data Handling and Preliminary Analysis

The study examined missing data patterns using Little's (1988) MCAR test, which was non‐significant (*p* = 0.214), indicating that the data were Missing Completely at Random (MCAR). This result suggests that data imputation was not strictly necessary (Little's 1988). However, to maintain data consistency and retain all cases for subsequent analyses, the Expectation Maximisation algorithm was applied to handle this negligible proportion of missing values (i.e., 116 total cases; 0.46%). The IBM Statistical Package for the Social Sciences (SPSS) version 28 was utilised for all analyses, except for the Rasch analysis, which employed RUMM2030. Since our sensitivity analyses showed that the selected subsample mirrored the characteristics of the full dataset, the Rasch analysis was conducted using this subsample only, without additional testing of the hold‐out data, to avoid redundancy. Notably, sensitivity analyses were conducted using random subsampling procedures. Specifically, the inter‐item correlation matrix derived from a random subsample of 1000 participants was compared with that obtained from the full sample to assess the stability and consistency of the observed correlation structure. Consistency was evaluated based on the similarity in the direction and magnitude of corresponding inter‐item correlations, with most coefficients differing only marginally (generally within approximately ±0.08), and the overall positive and negative affect item clustering patterns remaining stable across samples. This stability demonstrates that the Rasch model generalised well to the full sample (cf. Adu et al. 2025, 2026). Moreover, the Rasch‐based ordinal‐to‐interval transformation derived from the calibration sample was subsequently applied to the total dataset for all further analyses, ensuring that the full sample was represented in the results. Analyses in the current study included Pearson's correlation, paired samples *t*‐test, descriptive statistics, and psychometric evaluations. The utilisation of Rasch‐transformed interval scores for all variables enabled us to apply the required parametric test tools in our analyses.

#### 2.4.2 Rasch Model Evaluation

An initial likelihood ratio test conducted in RUMM2030 was significant (*p* < 0.001), rejecting the rating scale model and confirming that the unrestricted Partial Credit Rasch Model was appropriate for parameter estimation in the present analysis (Masters 1982). This model accounts for variations in item difficulty and respondents' ability levels, without presuming uniformity of these characteristics across items. By permitting item‐specific adjustments, the Partial Credit Model enhances both the overall measurement scale and the performance of individual items (Medvedev et al. 2020; Tennant and Küçükdeveci 2023). For assessing the overall fit of the data to the Rasch model, the study initially examined the *χ* ^*2*^ test for item‐trait interaction. The analysis aimed for a non‐significant result (*p* > 0.05) to indicate a good fit. Additionally, the residual values fell within an acceptable range of −2.50 to +2.50. The study also examined the residual correlations between items and ensured that they were below 0.20 (Tennant and Küçükdeveci 2023). In terms of unidimensionality, the study considered the lower bound of confidence intervals, which should align with 5%. Furthermore, ≤ 5% of cases should show potential for measuring multiple dimensions (Hagell 2015).

To evaluate threshold ordering, the study examined the Item Characteristic Curves (ICCs) and aimed to achieve monotonous patterns of items (Andrich 1978). Sample targeting was assessed by examining the item mean, which should fall between +0.50 and −0.50, with a mean of 0.00. Any item local dependence, indicated by residual correlation matrices above 0.20, was addressed using the advanced testlets creation methodology (Tennant and Küçükdeveci 2023). The study estimated the reliability of the scale using the PSI. To evaluate measurement invariance across different sociodemographic factors, the study used the Differential Item Functioning (DIF; Hagquist and Andrich 2017). For age group invariance, the study categorised age into groups (18–29 years, 30–46 years, and 47–89 years) based on percentiles.

DIF was investigated through ANOVA and Bonferroni‐adjusted pairwise comparison *t*‐tests to identify potential item bias across the sociodemographic factors. DIF analyses were performed using Bonferroni‐adjusted *p*‐values to account for multiple testing across items and grouping variables, thereby maintaining a conservative Type I error rate. The study also used the final person measure to develop an algorithm to transform ordinal data into interval‐level data (Medvedev et al. 2020). For a detailed description of the estimation procedures, readers are referred to Medvedev and Krägeloh (2025). The study conducted a paired sample *t*‐test to determine if there was a statistical difference between the scores at the ordinal and interval levels. Finally, the study assessed convergent and discriminant validity using Pearson's correlation coefficients.

## 3 Results

The initial analysis (Table 1) revealed a statistically significant *χ* ^*2*^ resulting from the interaction between the individual items and the latent variable, indicating that the data failed to fit the overall Rasch model for both the PA (*χ* ^2^ (40) = 361.15, *p* < 0.001) and NA (*χ* ^2^ (40) = 94.28, *p* < 0.001) subscales. Unidimensionality evidence was also not present for the PA subscale (confidence interval lower bound of 5.3%), but it was present for the NA subscale (confidence interval lower bound at 1.0%). The reliabilities were found to be very good for PA (PSI = 0.88) and NA (PSI = 0.84). The study combined locally dependent items (i.e., items with residual correlations above the threshold of 0.20) into testlets, resulting in 4 testlets for the PA subscale (items: 1 + 6, 2 + 10, 3 + 9, 5 + 8; Table 2) and 5 testlets for the NA subscale (items: 11 + 17, 12 + 20, 13 + 14, 16 + 19, 15 + 18; Table 2). This procedure successfully resolved the issue of model misfit for the NA subscale only, indicating an overall satisfactory model fit (Table 1, A2: *χ* ^2^ (45) = 47.29, *p* = 0.380). Evidence of unidimensionality was observed, with a confidence interval lower bound of 2.9%, along with improved reliability (PSI = 0.87).

**TABLE 1:** Rasch model fit statistics for the initial and final analysis of the PANA (*n* = 1000).

	Item fit residual		Person fit residual		Goodness of fit			Unidimensionality *t‐*test	
Analyses (A)	Mean SD		Mean SD		*χ* ^2^ (df)	*p*	PSI	%	Lower bound
Positive affect									
A1 Initial	0.14	4.76	−0.57	1.84	361.15 (40)	< 0.001	0.88	6.7	5.3 (NO)
A2 testlet1	0.31	1.61	−0.50	1.39	63.45 (30)	< 0.001	0.90	3.7	2.3 (YES)
A3 Final	0.17	0.80	−0.55	1.11	19.43 (18)	0.37	0.91	3.1	1.7 (YES)
Negative affect									
A1 Initial	0.23	1.66	−0.41	1.52	94.28 (40)	< 0.001	0.84	2.4	1.0 (YES)
A2 Final	0.13	1.66	−0.43	1.16	47.29 (45)	0.38	0.87	4.3	2.9 (YES)

Abbreviation: PSI, Person separation index without extremes.

**TABLE 2:** Items fit statistics including the initial and final analyses of the PANA (*n* = 1000).

Item no.	Item content	Location	Fit residual	*χ* ^*2*^
Positive affect				
1	Interested	−0.28	3.62	21.51*
3	Excited	−0.26	6.30	92.55*
5	Strong	−0.01	−2.40	18.85*
9	Enthusiastic	0.36	−3.94	40.30*
10	Proud	0.22	8.46	60.70*
12	Alert	0.12	1.54	3.71
14	Inspired	0.22	−5.89	40.28*
16	Determined	−0.05	−3.12	35.76*
17	Attentive	−0.36	0.23	13.28
19	Active	0.03	−3.40	34.21*
Initial Testlets (T) creation				
T1	Items: 1 + 12	−0.18	2.70	6.31
T2	Items: 3 + 19	−0.19	−0.71	10.29
T3	Items: 5 + 17	−0.24	−1.12	7.64
T4	Items: 10 + 16	0.03	1.77	11.09
4	9	0.38	0.38	14.62
7	14	0.21	−1.15	13.50
Final Testlets (T) creation				
T1	T1 + T4	0.00	0.63	2.59
T2	T2 + 4	0.03	0.63	5.98
T3	T3 + 7	−0.02	−0.75	10.87
Negative affect				
2	Distressed	−0.29	3.57	10.48
4	Upset	−0.26	−0.22	7.82
6	Guilty	0.06	−0.58	12.84
7	Scared	0.37	0.81	9.48
8	Hostile	0.27	1.79	5.60
11	Irritable	−0.15	0.83	5.00
13	Ashamed	0.28	−1.82	19.27*
15	Nervous	−0.32	−1.50	13.88
18	Jittery	−0.18	0.74	1.16
20	Afraid	0.22	−1.30	8.76
Testlets (T) creation				
T1	Items: 2 + 13	−0.01	0.25	8.69
T2	Items: 4 + 20	0.01	−1.80	10.29
T3	Items: 6 + 7	0.18	0.16	8.43
T4	Items: 11 + 18	−0.24	2.71	9.70
T5	Items: 8 + 15	0.06	−0.68	10.18

However, although there was improvement in the model parameters for the PA subscale, the overall model fit was not evident (see Table 1, A2; *χ* ^2^ (30) = 63.45, *p* < 0.001). For instance, unidimensionality was achieved with a confidence interval lower bound of 2.3%, and reliability was excellent (PS1 = 0.90). Further modifications to the model, specifically combining teslet 1 with testlet 4, teslet 2 with item 4, and testlet 3 with item 7, led to the achievement of a good overall model fit for the PA subscale (Table 1, A3: *χ* ^2^ (18) = 19.43, *p* = 0.370). This resulted in obtaining strict unidimensionality with confidence interval lower bound of 1.7% and excellent reliability (PSI = 0.91). At this stage, it was found that response threshold categories worked appropriately for all items for all the scales. This was indicated by the monotonous patterns in the ICCs for all items (Supporting Information S1). Table 2 provides an overview of the individual item fit statistics within the Rasch model, including item locations, fit residuals, and *χ* ^*2*^ parameters.

### 3.1 DIF, Person‐Item Trait, and Ordinal‐To‐Interval Conversion

**FIGURE 1:**
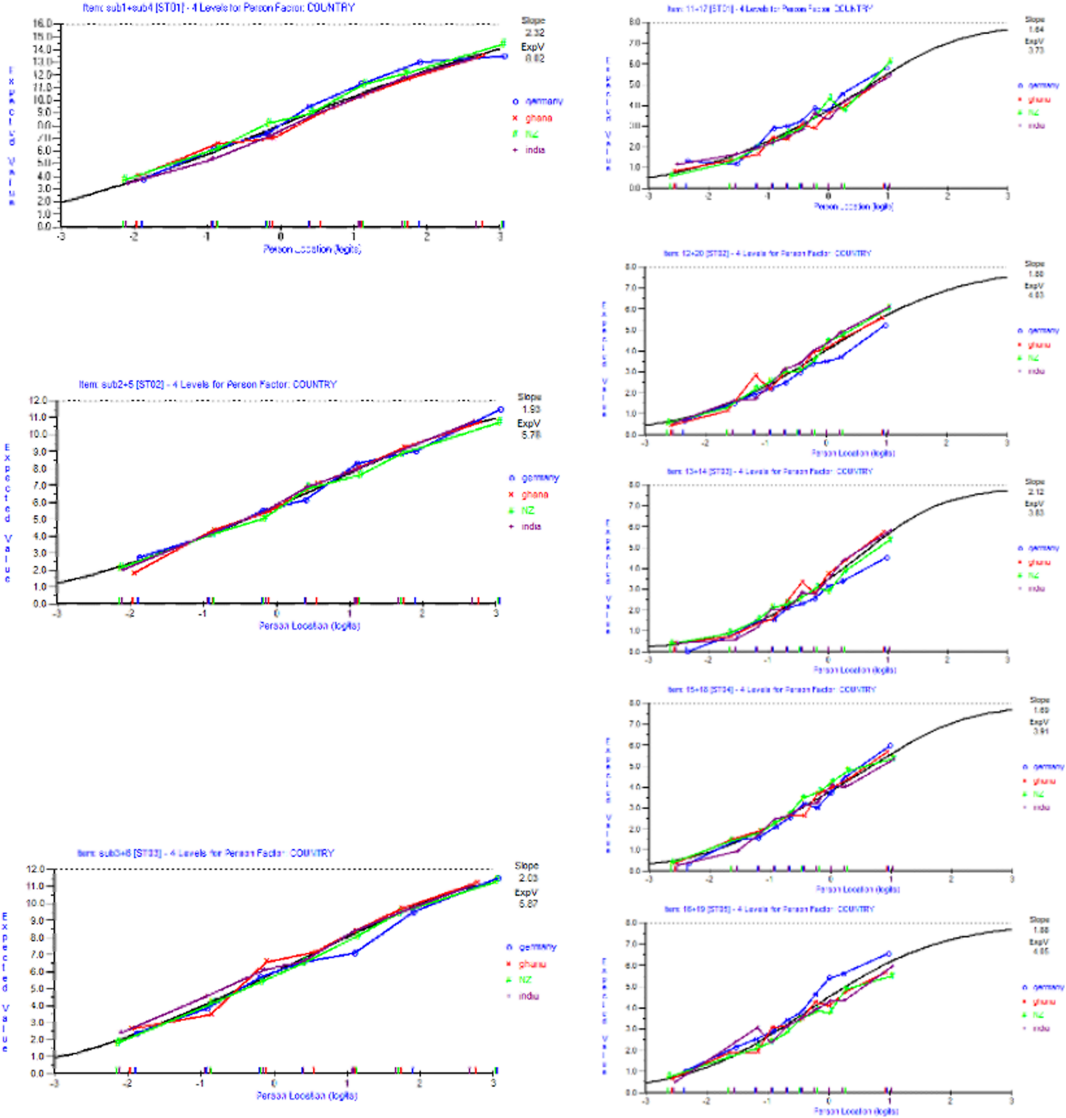
Differential item functioning (DIF) curves of the PA (left) and NA (right) subscales for country.

**FIGURE 2:**
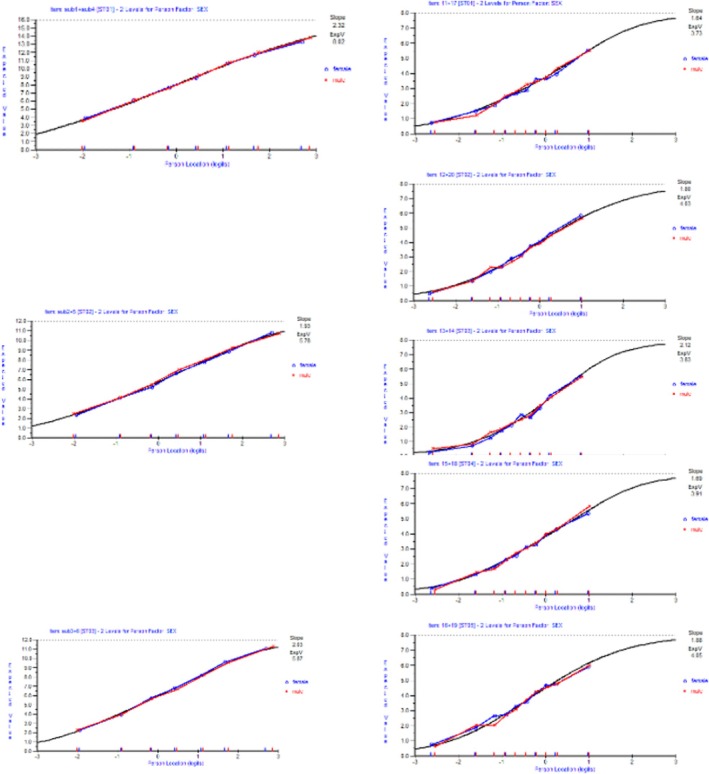
Differential item functioning (DIF) curves of the PA (left) and NA (right) subscales for participants' biological sex.

**FIGURE 3:**
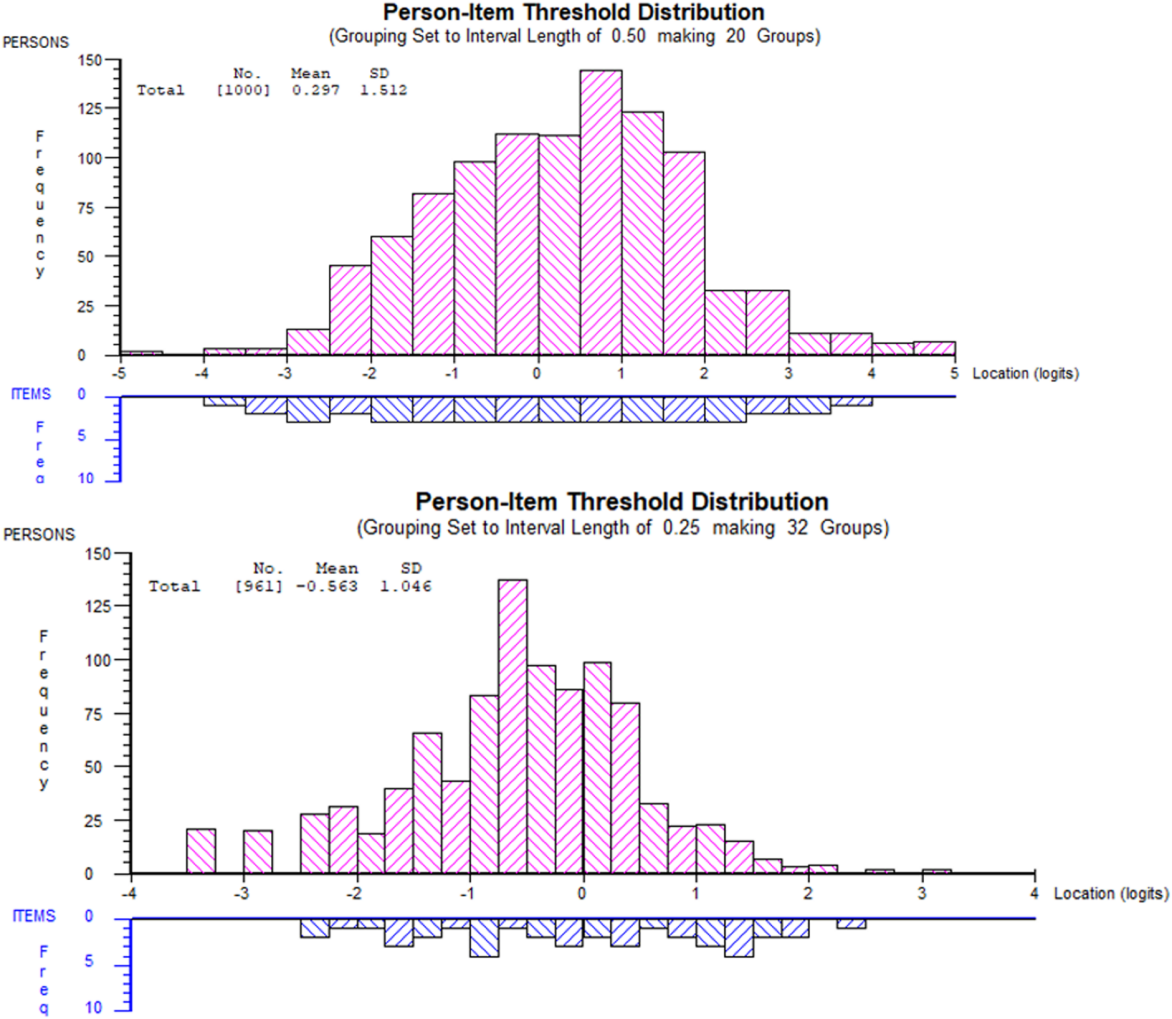
Person‐item threshold distributions for the PA (top) and NA (bottom) subscales.

Additionally, items did not show any evidence of DIF for participants regarding their country of origin (Figure 1), sex (Figure 2), being either health worker or from the general population (Supporting Information S1), and their education levels (Supporting Information S1). The person‐item distribution thresholds showed that both the PA (*M* = 0.30, SD = 1.51) and NA (*M* = −0.56, SD = 1.05) subscales effectively targeted the sample, showing no significant ceiling or floor effects (Figure 3). The study then developed an algorithm based on the person measures to convert ordinal scores into interval‐level data (Table 3). A paired samples *t*‐test revealed significant differences within the means of the ordinal scores for the PA (*M* = 33.13, SD = 8.22; *t*(1821) = −9.71, *p* < 0.001) and NA (*M* = 22.98, SD = 8.69); *t*(1821) = 40.27, *p* < 0.001 subscales compared to the Rasch‐transformed interval scores for the PA (*M* = 30.28, SD = 5.98) and NA (*M* = 25.62, SD = 6.86) subscales.

**TABLE 3:** Ordinal‐to‐interval transformation for the PANAS raw scores in logits and the original scale metric.

Positive affect			Negative affect		
	Interval			Interval	
Ordinal scores	Logits	Scale	Ordinal scores	Logits	Scale
10	−4.94	10.00	10	−4.09	10.00
11	−4.17	13.14	11	−3.31	13.92
12	−3.63	15.36	12	−2.78	16.57
13	−3.25	16.92	13	−2.43	18.35
14	−2.94	18.18	14	−2.15	19.73
15	−2.68	19.27	15	−1.93	20.86
16	−2.44	20.24	16	−1.73	21.83
17	−2.22	21.14	17	−1.56	22.71
18	−2.02	21.99	18	−1.40	23.50
19	−1.82	22.80	19	−1.26	24.24
20	−1.63	23.57	20	−1.12	24.93
21	−1.45	24.33	21	−0.99	25.59
22	−1.27	25.06	22	−0.86	26.22
23	−1.09	25.78	23	−0.74	26.84
24	−0.92	26.48	24	−0.62	27.44
25	−0.75	27.18	25	−0.50	28.03
26	−0.59	27.86	26	−0.39	28.61
27	−0.42	28.53	27	−0.28	29.17
28	−0.26	29.19	28	−0.16	29.74
29	−0.10	29.84	29	−0.05	30.30
30	0.06	30.50	30	0.06	30.85
31	0.21	31.14	31	0.17	31.39
32	0.37	31.78	32	0.27	31.94
33	0.53	32.43	33	0.38	32.49
34	0.68	33.07	34	0.49	33.03
35	0.84	33.71	35	0.60	33.56
36	0.99	34.35	36	0.70	34.11
37	1.15	35.01	37	0.81	34.65
38	1.31	35.66	38	0.92	35.21
39	1.48	36.33	39	1.03	35.77
40	1.64	37.01	40	1.15	36.35
41	1.81	37.70	41	1.27	36.95
42	1.99	38.43	42	1.40	37.59
43	2.17	39.20	43	1.53	38.28
44	2.37	40.02	44	1.68	39.04
45	2.60	40.93	45	1.85	39.89
46	2.85	41.96	46	2.05	40.88
47	3.14	43.17	47	2.29	42.11
48	3.52	44.71	48	2.62	43.74
49	4.05	46.89	49	3.11	46.22
50	4.80	50.00	50	3.86	50.00

*Note*: This conversion table can only be used for complete responses to the 10 items of each subscale. To use this table, ordinal raw scores (left column) of each subscale should be obtained by adding the observed scores for all 10 items. Next, match the ordinal total score (10–50) to the corresponding interval score in the right column (scale: 10–50). A final converted score between 10 and 50 will be obtained, with higher scores on the PA and NA subscales corresponding to higher favourable and unfavourable emotions, respectively.

### 3.2 Convergent and Discriminant Validity

#### 3.2.1 Ordinal Scores

The ordinal scores showed that NA significantly negatively correlated with self‐compassion (*r* = −0.40, *p* < 0.001), compassion towards others (*r* = −0.16, *p* < 0.001), and optimism (*r* = −0.36, *p* < 0.001), but a positive correlation with psychological distress (*r* = 0.53, *p* < 0.001). Conversely, PA correlated positively with self‐compassion (*r* = 0.38, *p* < 0.001), compassion towards others (*r* = 0.37, *p* < 0.001), and optimism (*r* = 0.38, *p* < 0.001), while correlating negatively with psychological distress (*r* = −0.32, *p* < 0.001). PA and NA were inversely linked but not significantly related (*r* = −0.04, *p* = 0.136).

#### 3.2.2 Interval Scores

Using the Rasch‐transformed interval scores, PA significantly positively correlated with compassion towards others (*r* = 0.36, *p* < 0.001), self‐compassion (*r* = 0.28, *p* < 0.001), and optimism (*r* = 0.36, *p* < 0.001) and negatively associated with psychological distress (*r* = −0.30, *p* < 0.001). NA correlated positively with distress (*r* = 0.53, *p* < 0.001) and negatively with self‐compassion (*r* = −0.33, *p* < 0.001), optimism (*r* = −0.39, *p* < 0.001), and compassion towards others (*r* = −0.15, *p* < 0.001). There was no significant association between NA and PA for either the interval scores (*r* = −0.04, *p* = 0.108) or the ordinal scores (*r* = −0.04, *p* = 0.136).

## 4 Discussion

This study evaluated the psychometric statistics of the PANAS using the Rasch model across four countries. The initial model showed misfitting indices; however, after employing the testlet creation methodology to modify the model, the study achieved acceptable model fits for both the PA and NA subscales. The scales were also found to be strictly unidimensional, with sound reliabilities. There was evidence of measurement invariance across various sociodemographic factors, as well as convergent and discriminant validities for both scales. Items showed good targeting and a monotonous pattern. To enhance the measurement precision of both scales, the study developed an algorithm to transform ordinal data into interval‐level scores. The initial model misfit observed in our results suggests that the observed response patterns deviated from those expected under the Rasch model, that is, the relationship between item difficulty and person ability did not fully conform to the model's probabilistic expectations (Boone 2016). Such deviations may result from local item dependencies, multidimensionality, or response biases that violate the model's assumptions. This misfit was resolved with the testlet creation approach (Tennant and Küçükdeveci 2023).

Testlet modification strategies combine items with similar content into more meaningful items, reducing measurement error and capturing unique aspects of the underlying latent variable. Items that share similar content in psychometric assessment become redundant, resulting in decreased measurement efficiency without contributing to the construct validity of the scale. This could adversely affect a scale's model fit, as seen in our initial fit indices (Tennant and Küçükdeveci 2023). The fact that model fit was resolved by creating testlets shows the methodological merits of this approach (Tennant and Küçükdeveci 2023). The improvement in model fit following testlet creation also implies that these dependencies reflected measurement artefacts rather than conceptual divergence. From a practical perspective, the use of testlets ensures that the resulting person estimates are not artificially inflated due to item redundancy, reflecting genuine trait differences rather than artefacts of item interdependence. This enhances the reliability and validity of the scale scores. Importantly, the construct being measured remains the same, preserving the conceptual integrity of the scale.

Testlet formation does not alter the substantive interpretation of the scale but rather refines its psychometric precision by controlling for item redundancy. This approach strengthens the scale's unidimensionality and interpretability, ensuring that score comparisons and subsequent analyses more accurately reflect the underlying latent trait (Medvedev and Krägeloh 2025; Tennant and Küçükdeveci 2023). The recreation of testlets by combining standalone items with existing testlets, particularly within the PA subscale, indicated that some individual items remained locally dependent on items already grouped within testlets. This suggested that the initial clustering did not fully account for shared variance. The restructured testlets ensured that these dependencies were adequately modelled, thereby improving overall model fit and maintaining measurement precision. This decision was data‐driven, guided by empirical evidence rather than item content, and aimed at improving overall model fit and construct validity.

Notably, all testlet creation in this study was based solely on statistical indicators, specifically, residual correlations exceeding 0.20, since relying on item content can be misleading (Boateng et al. 2018). Contextual or methodological factors, rather than semantic similarity, often drive such local dependencies (Boateng et al. 2018; Christensen et al. 2017; Tennant and Conaghan 2007). Moreover, while item content may suggest theoretical groupings, these do not necessarily reflect the actual error variance or dependencies within the data. Statistical analyses provide empirical evidence of how items interact, uncovering the underlying dependencies that are truly embedded within the data. Additionally, testlets can only be reliably created when the actual error variance is empirically known. Therefore, relying on statistical criteria ensured a more accurate and objective basis for grouping items into testlets. Essentially, the testlets benefit the interval‐level scores.

Researchers are encouraged to exercise caution when using testlets, as testlets can sometimes obscure rather than resolve item dependencies, particularly when applied arbitrarily (Christensen et al. 2017; Marais and Andrich 2008). In the present study, testlet formation was guided by empirical evidence of shared residual variance. Specifically, items were grouped only when residual correlations exceeded 0.20, indicating statistically meaningful local dependence. This data‐driven approach ensured that testlets modelled true shared variance rather than artificially masking it, thereby preserving the integrity of the latent construct while enhancing overall model fit. Similarly, Medvedev et al. (2023) also utilised the testlet methodology to obtain optimal model fit indices for both the PA and NA subscales. They additionally confirmed unidimensionality for each scale, like ours. The presence of unidimensionality indicates that a single score is appropriate for evaluating the underlying overall latent variable (i.e., PA or NA).

Contrasting with previous studies, Peter et al. (2016) reported that the PA subscales may not be measuring a single underlying latent variable of positive affect. These single scores, or person measures, accurately reflect individuals' levels on the latent variable, as scores reflect the difficulty of the items and individuals' ability. This explains the concepts of good targeting and the monotonous patterns found in the item threshold category. In simple terms, items on either of the scales can discriminate individuals who are low and high on the latent variable, congruence with the findings of Medvedev et al. (2023) and Pires et al. (2013). Notwithstanding, Peter et al. (2016) results showed a floor effect for the NA subscale, meaning a significant number of participants in their study scored the lowest possible score on the NA subscale.

The current Rasch‐based reliabilities focus on individual response patterns. They provide a detailed reliability estimate that reflects how well each item contributes to measuring the underlying latent variable for each individual respondent. This approach allows for a more nuanced understanding of measurement reliability compared to the traditional methods like the CFA. The CFA typically assesses reliability at the population level using inter‐item correlations, which may not capture individual variations in the same way that Rasch analysis does. The current reliability coefficients for both the PA and NA subscales met the criteria for them to be used for both individual and group‐level assessment (Fisher Jr 1992).

The reliability and validity of the scores is enhanced when utilising the interval‐level scores of the PA and NA subscales developed by the current researchers in assessments. For example, if the PA score of a person increases from 10 to 15 in an intervention, while in the other, it rises from 15 to 20. Although these changes might appear equal in an ordinal scoring system, the change in the first scenario is 9.27 units, compared to 4.3 units in the second scenario using the interval scores (Table 3). This emphasises the significance of using Rasch interval scores, as they can offer nuances in measurements and provide clarity on actual changes in measures among individuals in both research and clinical contexts (Tennant and Küçükdeveci 2023). This implies that these measures can effectively capture subtle differences in an individual's affective states. Another advantage of using the interval‐level scores developed by the current researchers is their reduced measurement error compared to ordinal scores. Essentially, the interval scores can be directly applied without additional validation. Researchers and practitioners simply obtain the total ordinal score and use the conversion table to retrieve the corresponding interval score, saving time and enabling precise, comparable measurement across studies and applications.

The evidence of measurement invariance across the sociodemographic factors implies that the measurement properties of both the PA and NA subscales are consistent and reliable across different groups and characteristics, consistent with previous research (Tennant and Küçükdeveci 2023; Medvedev et al. 2023). This suggests that the scale measures the positive and negative effects in a consistent manner, regardless of variations in individual characteristics such as age, gender, educational level, and country of origin (Medvedev and Krägeloh 2022). Our study offers the strength of providing robust evidence of measurement invariance, particularly due to the assessment of numerous personal factors. This evidence enables confident comparisons between studies, facilitating reviews studies (Welzel et al. 2023). This property of our study also increases its external validity typically stemming from the use of four distinct countries across four continents.

Further evidence supporting the validity of the scales was found through discriminant and convergent validity findings. The PA subscales showed a positive association with measures of self‐compassion, optimism, and compassion towards others. These results provide evidence of convergent validity for the PA subscales and support previous studies that identify these variables as protective factors for positive emotions (Förster and Kanske 2022). On the other hand, the NA subscale demonstrated convergent validity by positively correlating with psychological distress. This also aligns with the literature suggesting that distress is a risk factor for negative affect. The weak non‐significant correlation between the PA and NA subscales indicated their discriminant validity, showing that the scale measures a construct distinct from related but theoretically different domains.

Additionally, the weak negative associations observed between the PA subscale and measures of psychological distress, as well as between the NA subscale and measures of self‐compassion, optimism, and compassion towards others, support the hypothesis of discriminant validity. These findings further reflect theoretically consistent inverse relationships between conceptually distinct constructs (Guan et al. 2021). The study also showed that self‐compassion, optimism, and compassion towards others served as protective factors for positive affect, which in turn helped protect against distress. Conversely, distress acted as a risk factor for negative affect, while self‐compassion, optimism, and compassion towards others also served as protective factors against negative affect. This evidence has been consistently reported in the existing literature (Guan et al. 2021).

Notably, a comparison of the correlation matrices revealed important insights. A consistent pattern emerged across both analyses, showing that correlations based on interval‐level scores tended to exhibit reduced and non‐significant links compared to those using ordinal scores. In ordinal scales (e.g., Likert 1–5), the distances between response options are not truly equal; the difference between ‘1’ and ‘2’ may not represent the same change in the underlying trait as between ‘4’ and ‘5’ (Boone 2016). However, traditional analyses such as Pearson correlations treat these categories as equally spaced, which can make associations appear artificially stronger. The Rasch transformation corrects for this by assigning scores based on the actual probabilistic distances between response categories, producing more precise, though sometimes smaller, correlations (Boone 2016).

Rasch modelling also separates true latent trait variance from measurement error and item bias, so interval scores retain only the ‘true’ variance (Medvedev and Krägeloh 2025; Tennant and Küçükdeveci 2023). Consequently, shared random noise that may inflate ordinal correlations, especially among weakly related constructs such as NA and compassion towards others, is reduced. For instance, in the current study, the weak negative correlation between NA and compassion towards others (*r* = −0.16) in the ordinal data likely reflected method variance or general response tendencies (e.g., positivity bias). After Rasch scaling removed this error variance, the true association appeared negligible, resulting in a non‐significant correlation, thus reflecting a more accurate estimate of the relationship between constructs. This further shows the robustness of using the interval scores. The significant differences between the ordinal and Rasch‐transformed interval scores further indicate that the two scoring approaches are not directly interchangeable. Collectively, these differences primarily reflect the recalibration of ordinal raw scores into interval‐level estimates under the Rasch model, thereby improving measurement precision and reducing measurement artefacts, rather than indicating substantive changes in the underlying constructs being assessed.

Notwithstanding, the discrepancies in the dimensionality of the PANAS across the Rasch‐based studies may stem from sex and cross‐cultural differences in perceiving, understanding, and expressing affective states. For example, in traditional masculine cultures, men expressing negative emotions like sadness may be misconstrued as a sign of weakness (Fischer and Manstead 2000). Moreover, in individualist cultures such as the US, emotions are often experienced in a polarised manner, whereas in collectivist cultures like China, emotions are seen as interconnected and dialectical (Bagozzi et al. 1999). These sociocultural factors can complicate the accurate cross‐cultural measurement of affective states (Pires et al. 2013). Such pitfalls may have resulted from the fact that most Rasch‐based studies, including the current one, used homogeneous samples. These samples may not capture the full range of variability in responses that a more diverse sample would.

Although the present study employed different recruitment approaches to enable broad participation across diverse contexts, this may have introduced variability in sample characteristics. While this was addressed with DIF analyses across demographic groups, the heterogeneity in recruitment methods may limit the generalisability of the findings. Thus, adapting the PANAS to incorporate culturally specific expressions of affect and implementing more focused and consistent sample recruitment strategies (e.g., representative samples) across cultures could improve its universal acceptability and enhance well‐being outcomes. Typically, developing translated versions of the scale in local dialects across Ghana and India could strengthen the assessment of culturally nuanced affective experiences. While RUMM2030 permits testing DIF primarily through ANOVA and Bonferroni‐adjusted pairwise *t*‐tests, this approach is both methodologically sound and widely accepted in contemporary Rasch practise (Andrich 1978; Medvedev and Krägeloh 2025). It effectively detects both uniform and non‐uniform DIF while maintaining control over Type I error rates. Nonetheless, triangulating these findings with alternative methods, such as the Mantel–Haenszel *χ* ^*2*^ test, could offer additional evidence of item invariance and further strengthen the robustness of the PANAS cross‐group comparisons. Finally, further studies are required to test the external validity of Rasch‐based scores.

## 5 Conclusions

The present study utilised community samples from four countries to evaluate the psychometric statistics of the PANAS. Results demonstrated that both the PA and NA subscales are reliable and valid for assessing positive and negative affect across these countries, regardless of individuals' sociodemographic factors. The interval‐level scores developed by the current researchers can enhance the measurement precision of both scales. Self‐compassion, optimism, and compassion towards others protected positive affect and reduced distress, whereas distress acted as a risk factor for negative affect. This study also acknowledges the significant sociocultural impact on the expression of affect and advocates for more careful translations of the PANAS to further improve its universal acceptability, thereby promoting health and well‐being. However, most Rasch studies, including the current one, demonstrate internal psychometric soundness but stop short of testing the external validity of scores. There is also the potential for testlets to obscure item dependencies if not empirically justified. The variability in recruitment methods across the countries may limit cross‐cultural generalisability. Triangulating the current findings with alternative methods such as the Mantel–Haenszel *χ* ^*2*^ would further reinforce the robustness of the scale's invariance across demographic groups.

## Author Contributions

**Peter Adu:** conceptualization, methodology, investigation, writing – original draft, writing – review and editing, formal analysis, data curation.

## Ethics Statement

The study was approved by the Human Research Ethics Committee of Victoria University of Wellington, New Zealand (#0000029770). The study was performed in accordance with the ethical standards as laid down in the 1964 Declaration of Helsinki and its later amendments. The study followed the standardised checklist for reporting cross‐sectional studies.

## Consent

Participants provided informed consent by clicking a button after reading the consent information. They agreed for their results to be published or used for academic purposes such as reports, presentations, and public documentation, with data presented in aggregate form (i.e., combined and analysed with others).

## Conflicts of Interest

The author declares no conflicts of interest.

## Supporting information


**Data S1:** Supporting Information.

## Data Availability

The data that support the findings of this study are available on request from the corresponding author. The data are not publicly available due to privacy or ethical restrictions.

## References

[ijop70230-bib-0040] Adu, P. 2025. “*Investigating Factors Associated With COVID‐19 Vaccination Attitudes* (Doctoral dissertation).” Open Access Te Herenga Waka‐Victoria University of Wellington.

[ijop70230-bib-0038] Adu, P. , T. Jurcik , and D. Grigoryev . 2021. “Mental Health Literacy in Ghana: Implications for Religiosity, Education and Stigmatization.” Transcultural Psychiatry 58, no. 4: 516–531. 10.1177/13634615211022177. 34165347

[ijop70230-bib-0039] Adu, P. , D. Miconi , and C. Rousseau . 2026. “Multi‐Method Cross‐Linguistic Validation of the Activism and Radicalism Intention Scale for Adolescents.” International Journal of Behavioral Development. 10.1177/01650254261423823.

[ijop70230-bib-0001] Adu, P. , T. Popoola , N. Iqbal , et al. 2025. “Cross‐Country Assessment of the Unique Contributions of Psychological Factors to Vaccination: Perspectives on the COVID‐19 Pandemic.” Health Psychology 30: 2385–2399. 10.1177/13591053241266592. PMC12322329 39081206

[ijop70230-bib-0037] Adu, P. , T. Popoola , A. Roemer , et al. 2023. “Validation and Cultural Adaptation of the Motors of COVID‐19 Vaccination Acceptance Scale (MoVac‐COVID19S) in German.” Psychological Test Adaptation and Development. 10.1027/2698-1866/a000064.

[ijop70230-bib-0002] Andrich, D. 1978. “A Rating Formulation for Ordered Response Categories.” Psychometrika 43: 561–573. 10.1007/BF02293814.

[ijop70230-bib-0003] Bagozzi, R. P. , N. Wong , and Y. Yi . 1999. “The Role of Culture and Gender in the Relationship Between Positive and Negative Affect.” Cognition & Emotion 13, no. 6: 641–672. 10.1080/026999399379023.

[ijop70230-bib-0036] Boateng, G. O. , T. B. Neilands , E. A. Frongillo , H. R. Melgar‐Quiñonez , and S. L. Young . 2018. “Best Practices for Developing and Validating Scales for Health, Social, and Behavioral Research: A Primer.” Frontiers in Public Health 6: 149. 10.3389/fpubh.2018.00149. 29942800 PMC6004510

[ijop70230-bib-0004] Boone, W. J. 2016. “Rasch Analysis for Instrument Development: Why, When, and How?” CBE—Life Sciences Education 15, no. 4: rm4. 10.1187/cbe.16-04-0148. 27856555 PMC5132390

[ijop70230-bib-0005] Brennan, D. , K. Singh , A. Spencer , and K. Roberts‐Thomson . 2006. “Positive and Negative Affect and Oral Health‐Related Quality of Life.” Health and Quality of Life Outcomes 4: 83. 10.1186/1477-7525-4-83. 17052358 PMC1626449

[ijop70230-bib-0006] Christensen, K. B. , G. Makransky , and M. Horton . 2017. “Critical Values for Yen's Q 3: Identification of Local Dependence in the Rasch Model Using Residual Correlations.” Applied Psychological Measurement 41, no. 3: 178–194. 10.1177/0146621616677520. 29881087 PMC5978551

[ijop70230-bib-0007] Fischer, A. H. , and A. S. Manstead . 2000. “The Relation Between Gender and Emotions in Different Cultures.” Gender and Emotion: Social Psychological Perspectives 1: 71–94.

[ijop70230-bib-0008] Fisher, W., Jr. 1992. “Reliability Statistics.” In Rasch Measurement Transactions, vol. 6, 238. MESA Press.

[ijop70230-bib-0009] Förster, K. , and P. Kanske . 2022. “Upregulating Positive Affect Through Compassion: Psychological and Physiological Evidence.” International Journal of Psychophysiology 176: 100–107. 10.1016/j.ijpsycho.2022.03.009. 35358613

[ijop70230-bib-0010] Glaesmer, H. , J. Hoyer , J. Klotsche , and P. Y. Herzberg . 2008. “Die deutsche version des Life‐Orientation‐Tests (LOT‐R) zum dispositionellen Optimismus und Pessimismus.” Zeitschrift für Gesundheitspsychologie 16, no. 1: 26–31. 10.1026/0943-8149.16.1.26.

[ijop70230-bib-0011] Guan, F. , Y. Wu , W. Ren , et al. 2021. “Self‐Compassion and the Mitigation of Negative Affect in the Era of Social Distancing.” Mindfulness 12: 2184–2195. 10.1007/s12671-021-01674-w. 34221182 PMC8236748

[ijop70230-bib-0012] Hagell, P. 2015. “Testing Unidimensionality Using the PCA/t‐Test Protocol With the Rasch Model: A Cautionary Note.” Rasch Measurement Transactions 28, no. 4: 1487–1489.

[ijop70230-bib-0013] Hagell, P. , and A. Westergren . 2016. “Sample Size and Statistical Conclusions From Tests of Fit to the Rasch Model According to the Rasch Unidimensional Measurement Model (Rumm) Program in Health Outcome Measurement.” Journal of Applied Measurement 17, no. 4: 416–431. 28009589

[ijop70230-bib-0014] Hagquist, C. , and D. Andrich . 2017. “Recent Advances in Analysis of Differential Item Functioning in Health Research Using the Rasch Model.” Health and Quality of Life Outcomes 15, no. 1: 1–8. 10.1186/s12955-017-0755-0. 28927468 PMC5606090

[ijop70230-bib-0015] Hemphill, J. F. 2003. “Interpreting the Magnitudes of Correlation Coefficients.” American Psychologist 58, no. 1: 78–79. 10.1037/0003-066X.58.1.78. 12674822

[ijop70230-bib-0016] Hwang, J. Y. , T. Plante , and K. Lackey . 2008. “The Development of the Santa Clara Brief Compassion Scale: An Abbreviation of Sprecher and Fehr's Compassionate Love Scale.” Pastoral Psychology 56: 421–428. 10.1007/s11089-008-0117-2.

[ijop70230-bib-0017] Klasko‐Foster, L. B. , S. Przybyla , H. Orom , E. Gage‐Bouchard , and M. T. Kiviniemi . 2020. “The Influence of Affect on HPV Vaccine Decision Making in an HPV Vaccine naïve College Student Population.” Preventive Medicine Reports 20: 101195. 10.1016/j.pmedr.2020.101195. 32983851 PMC7498828

[ijop70230-bib-0044] Kuppens, P. , A. Realo , and E. Diener . 2008. “The Role of Positive and Negative Emotions in Life Satisfaction Judgment Across Nations.” Journal of Personality and Social Psychology 95, no. 1: 66. 10.1037/0022-3514.95.1.66. 18605852

[ijop70230-bib-0018] Little, R. J. 1988. “A Test of Missing Completely at Random for Multivariate Data With Missing Values.” Journal of the American Statistical Association 83, no. 404: 1198–1202. 10.1080/01621459.1988.10478722.

[ijop70230-bib-0041] Lovibond, P. F. , and S. H. Lovibond . 1995. “The Structure of Negative Emotional States: Comparison of the Depression Anxiety Stress Scales (DASS) With the Beck Depression and Anxiety Inventories.” Behaviour Research and Therapy 33, no. 3: 335–343. 10.1016/0005-7967(94)00075-U. 7726811

[ijop70230-bib-0019] Marais, I. , and D. Andrich . 2008. “Effects of Varying Magnitude and Patterns of Response Dependence.” Journal of Applied Measurement 9, no. 2: 105–124. 18480508

[ijop70230-bib-0042] Masters, G. N. A. 1982. “Rasch Model for Partial Credit Scoring.” Psychometrika 47, no. 2: 149–174. 10.1007/BF02296272.

[ijop70230-bib-0020] Medvedev, O. N. , and C. U. Krägeloh . 2022. “Rasch Measurement Model.” In Handbook of Assessment in Mindfulness Research, 1–18. Springer.

[ijop70230-bib-0021] Medvedev, O. N. , and C. U. Krägeloh . 2025. “Rasch Measurement Model.” In Handbook of Assessment in Mindfulness Research, edited by R. J. Siegert , and N. N. Singh . Springer.

[ijop70230-bib-0022] Medvedev, O. N. , C. U. Krägeloh , E. A. Titkova , and R. J. Siegert . 2020. “Rasch Analysis and Ordinal‐To‐Interval Conversion Tables for the Depression, Anxiety and Stress Scale.” Journal of Health Psychology 25, no. 10–11: 1374–1383. 10.1177/13591053187552. 29402132

[ijop70230-bib-0023] Medvedev, O. N. , A. Roemer , C. U. Krägeloh , M. H. Sandham , and R. J. Siegert . 2023. “Enhancing the Precision of the Positive and Negative Affect Schedule (PANAS) Using Rasch Analysis.” Current Psychology 42, no. 2: 1554–1563. 10.1007/s12144-021-01556-3.

[ijop70230-bib-0024] Pelton, T. 2002. Where Are the Limits to the Rasch Advantage. International Objective Measurement Workshop.

[ijop70230-bib-0025] Peter, C. , S. E. Schulenberg , E. M. Buchanan , B. Prodinger , and S. Geyh . 2016. “Rasch Analysis of Measurement Instruments Capturing Psychological Personal Factors in Persons With Spinal Cord Injury.” Journal of Rehabilitation Medicine 48, no. 2: 175–188. 26926921 10.2340/16501977-2028

[ijop70230-bib-0026] Pires, P. , A. Filgueiras , R. Ribas , and C. Santana . 2013. “Positive and Negative Affect Schedule: Psychometric Properties for the Brazilian Portuguese Version.” Spanish Journal of Psychology 16: E58. 10.1017/sjp.2013.60. 24230921

[ijop70230-bib-0027] Pressman, S. D. , and S. Cohen . 2005. “Does Positive Affect Influence Health?” Psychological Bulletin 131, no. 6: 925–971. 16351329 10.1037/0033-2909.131.6.925

[ijop70230-bib-0028] Raes, F. , E. Pommier , K. D. Neff , and D. Van Gucht . 2011. “Construction and Factorial Validation of a Short Form of the Self‐Compassion Scale.” Clinical Psychology & Psychotherapy 18, no. 3: 250–255. 10.1002/cpp.702. 21584907

[ijop70230-bib-0029] Roemer, A. , and O. N. Medvedev . 2023. “Positive and Negative Affect Schedule (PANAS).” In Handbook of Assessment in Mindfulness Research, 1–11. Springer.

[ijop70230-bib-0030] Scheier, M. F. , C. S. Carver , and M. W. Bridges . 1994. “Distinguishing Optimism From Neuroticism (And Trait Anxiety, Self‐Mastery, and Self‐Esteem): A Reevaluation of the Life Orientation Test.” Journal of Personality and Social Psychology 67, no. 6: 1063–1078. 7815302 10.1037//0022-3514.67.6.1063

[ijop70230-bib-0043] Tennant, A. , and P. G. Conaghan . 2007. “The Rasch Measurement Model in Rheumatology: What Is It and Why Use It? When Should It Be Applied, and What Should One Look for in a Rasch Paper?” Arthritis Care & Research 57, no. 8: 1358–1362. 10.1002/art.23108. 18050173

[ijop70230-bib-0031] Tennant, A. , and A. A. Küçükdeveci . 2023. “Application of the Rasch Measurement Model in Rehabilitation Research and Practice: Early Developments, Current Practice, and Future Challenges.” Frontiers in Rehabilitation Sciences 4: 1208670. 10.3389/fresc.2023.1208670. 37529206 PMC10387545

[ijop70230-bib-0032] Tsai, J. L. , B. Knutson , and H. H. Fung . 2006. “Cultural Variation in Affect Valuation.” Journal of Personality and Social Psychology 90, no. 2: 288–307. 10.1037/0022-3514.90.2.288. 16536652

[ijop70230-bib-0033] Watson, D. , L. A. Clark , and A. Tellegen . 1988. “Development and Validation of Brief Measures of Positive and Negative Affect: The PANAS Scales.” Journal of Personality and Social Psychology 54, no. 6: 1063–1070. https://sid.ir/paper/631266/en. 3397865 10.1037//0022-3514.54.6.1063

[ijop70230-bib-0034] Welzel, C. , L. Brunkert , S. Kruse , and R. F. Inglehart . 2023. “Non‐Invariance? An Overstated Problem With Misconceived Causes.” Sociological Methods & Research 52, no. 3: 1368–1400. 10.1177/004912412199552.

[ijop70230-bib-0035] Young, K. S. , C. F. Sandman , and M. G. Craske . 2019. “Positive and Negative Emotion Regulation in Adolescence: Links to Anxiety and Depression.” Brain Sciences 9, no. 4: 76. 10.3390/brainsci9040076. 30934877 PMC6523365

