# Supplementary material for: Rasch Analysis and Interval‐Level Scaling of the Positive and Negative Affect Schedule (PANAS) Across Cultures
Source: Int J Psychol. 2026 Jun 8;61(4):e70230. doi: 10.1002/ijop.70230 (PMC13246275; doi:10.1002/ijop.70230)
Supplement: Supplementary file 1 — Data S1: Supporting Information. [file IJOP-61-e70230-s001.docx]

**Supplementary S1**

**
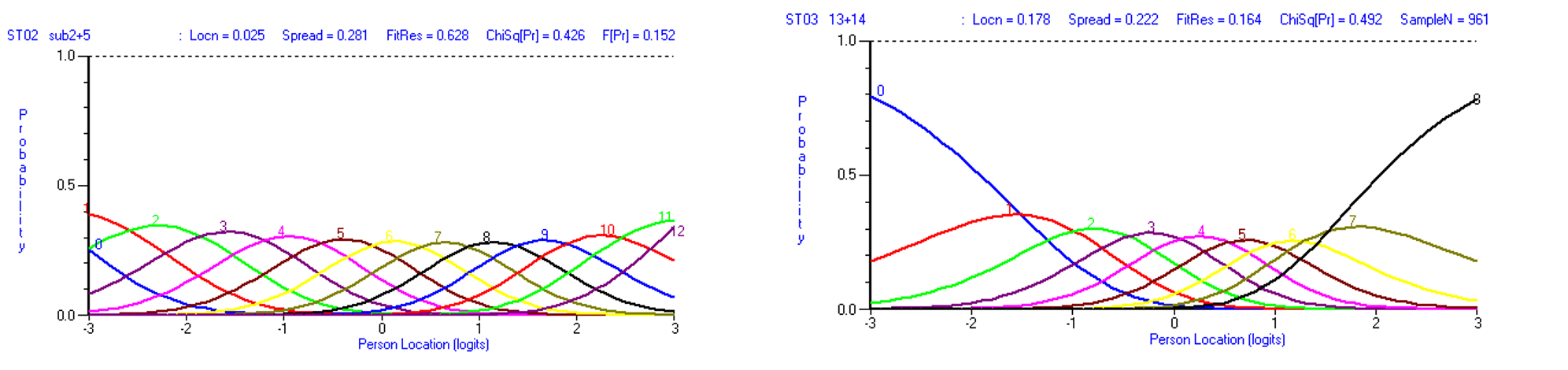
**

Item Characteristic Curve (ICC) for the final items of the PA and NA subscales. Example of ordered testlets for PA (bottom left) and NA (bottom right).


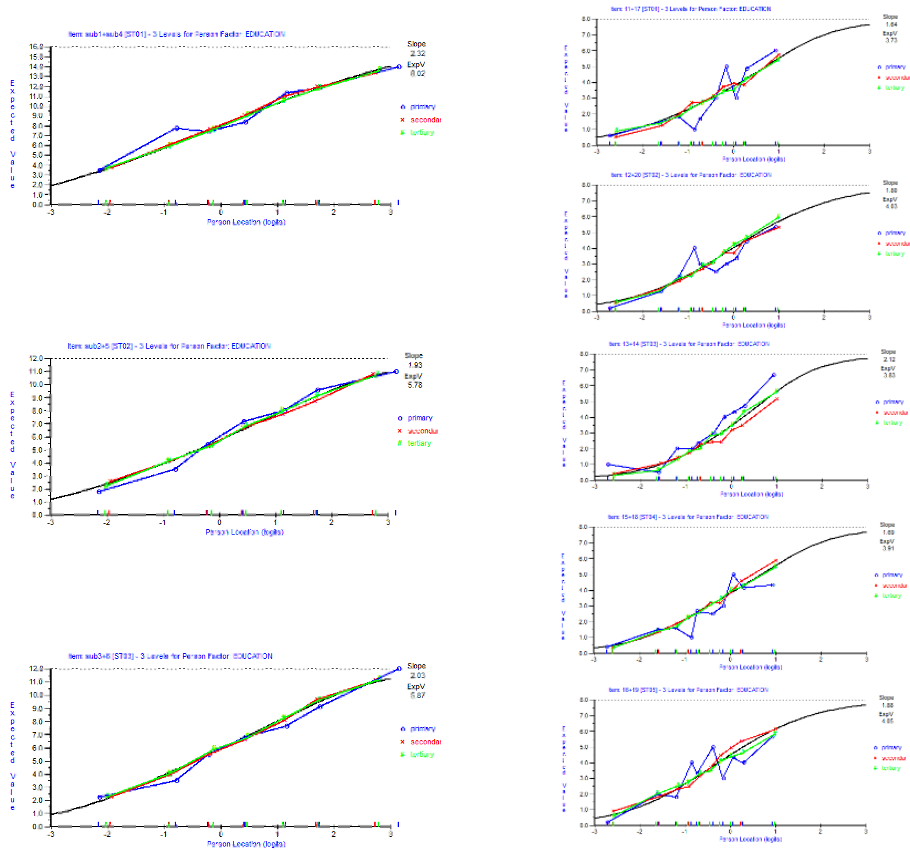


Differential Item Functioning (DIF) curves of the PA (left) and NA (right) subscales for age of the participants.

**
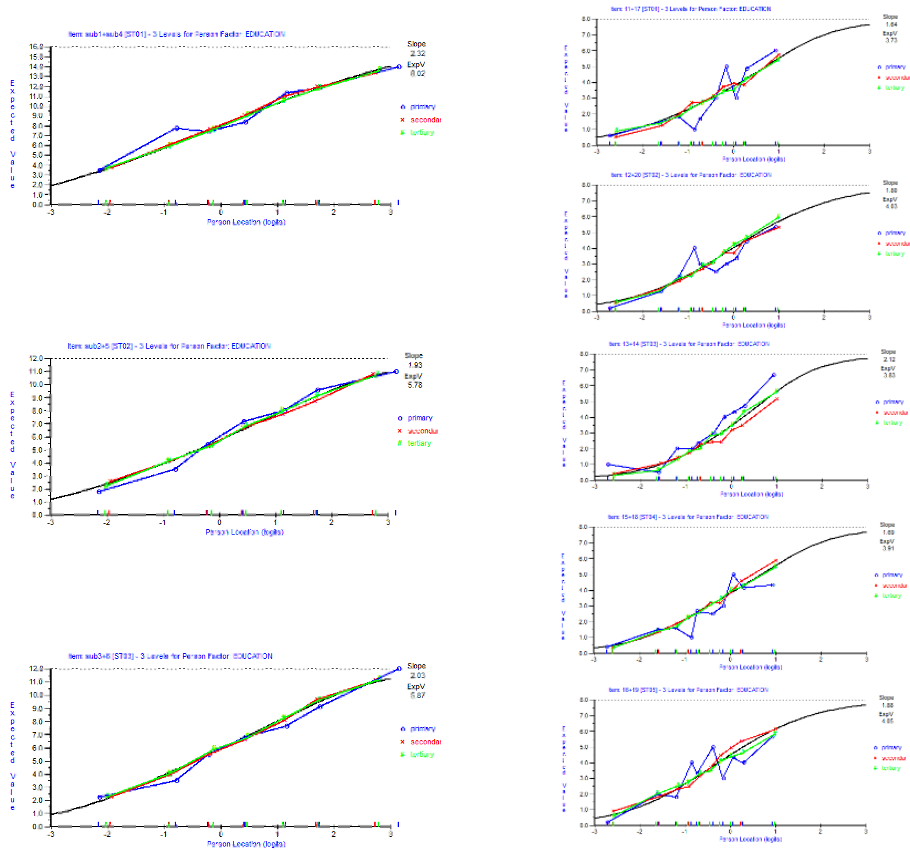
**

Differential Item Functioning (DIF) curves of the PA (left) and NA (right) subscales for educational levels of the participants.


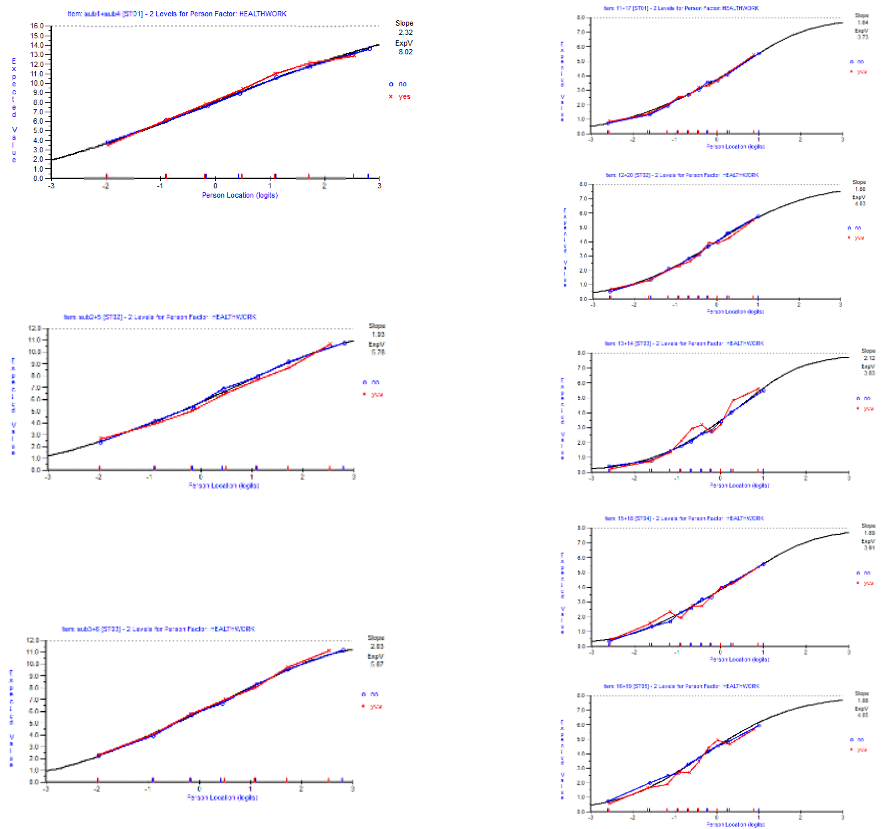


Differential Item Functioning (DIF) curves of the PA (left) and NA (right) subscales for health workers vs. general population within the participants
